# A novel deletion mutation in the *LPA* gene in a middle-aged woman with ischaemic stroke

**DOI:** 10.1186/s12920-021-00982-3

**Published:** 2021-05-18

**Authors:** Youran Li, Yizhong Wang, Fan Gong, Xiaofei Yu, Ting Zhang

**Affiliations:** 1 Department of Gastroenterology, Hepatology and Nutrition, Shanghai Children’s Hospital, Shanghai Jiao Tong University, Shanghai, 200062 China; 2 Department of Neurology, and Institute of Neurology, Shuguang Hospital Affiliated to Shanghai University of Traditional Chinese Medicine, Shanghai, 201203 China

**Keywords:** Mutation, *LPA*, Ischaemic stroke, Case report

## Abstract

**Background:**

Genetic diversity of the human *LPA* gene locus is associated with high plasma concentrations of lipoprotein(a) [Lp(a)]. High Lp(a) concentrations are strongly associated with a high incidence rate of ischaemic stroke.

**Case presentation:**

A 46-year-old female Chinese patient suffered from ischaemic stroke. Upon admission to the hospital, the patient was diagnosed with an elevated level of plasma Lp(a). The patient’s clinical symptoms were alleviated by administration of basilar artery stent thrombectomy, mannitol, and aspirin. A novel compound heterozygous deletion of the region containing exons 3–16 covering kringle IV copy number variation (KIV CNV) domains in the *LPA* gene was observed in genetic analysis by next-generation sequencing and confirmed by qPCR.

**Conclusions:**

In the current study, we reported a case of a 46-year-old female patient diagnosed with ischaemic stroke. This novel heterozygous deletion mutation in the *LPA* gene expands the spectrum of *LPA* mutations. Further study is required to understand the mechanism of *LPA* mutations in ischaemic stroke.

## Background

Human lipoprotein(a) [Lp(a)], a high polymer compound in plasma, is composed of one low-density lipoprotein (LDL) particle and one polymorphic glycoprotein named apolipoprotein(a) [apo(a)] [1]. Apo(a) is encoded by the *LPA* gene, which is located on chromosome 6q26-27 [2]. The human *LPA* gene is homologous to human plasminogen (*PLG*), which have high sequence similarity in coding regions of the kringle domains [2]. The human *PLG* gene has five different single copies of paralogous kringle domains (KI–KV), while KI–KIII is lost by deletion in the human *LPA* gene [3, 4]. There are 10 different types of KIV domains in the human *LPA* gene (KIV1–KIV10). All KIV-encoding domains consist of two exons that are present as single copies, except KIV2 [3]. The KIV2-encoding domain can have 1 to more than 40 copies in different populations, which is known as KIV2 copy number variation (CNV) [1]. The KIV2 CNV leads to high heterogeneity of the polypeptide chain length of apo(a), with molecular weights ranging from 300 to 800 kDa [5].

Human plasma concentrations of the Lp(a) lipoprotein vary substantially between different individuals, ranging from below 10 mg/L to more than 2000 mg/L [6]. Several studies have revealed that plasma Lp(a) level variation exists both in individuals of the same ancestry background and between different ancestries [7, 8]. For example, individuals of recent African ancestry have up to threefold higher plasma Lp(a) levels than white individuals, and women have 15–20% higher Lp(a) levels than men [9]. Although the exact mechanisms of Lp(a) level variation are incompletely understood, they have been associated with genetic variations in the *LPA* gene, including single-nucleotide variants and KIV2 CNV [10]. It has been reported that a reduced KIV2 copy number in *LPA* gene and a small Lp(a) lipoprotein size were closely related to an incremental level of Lp(a) lipoprotein [11].

Studies have demonstrated that high levels of Lp(a) are related to an increased risk of cardiovascular disease (CVD) due to its proatherosclerotic and prothrombotic properties [12, 13]. Recently, Sun et al. reported that the Lp(a) level and low KIV2 copy number are risk factors for coronary atherosclerotic heart disease (CAHD) in the Chinese Han population and that rising Lp(a) may determine the form of coronary plaque [14]. A meta-analysis showed that rising Lp(a) is an independent risk factor for ischaemic stroke [15]. Here, we report a middle-aged female who suffered ischaemic stroke with a novel compound heterozygous deletion covering the KIV2 CNV in the *LPA* gene from China. The clinical characteristics and genetic variations in *LPA* of the patient are described in this study.

## Case presentation

A 46-year-old Chinese female patient was admitted to the Department of Neurology at Shuguang Hospital Affiliated to Shanghai University of Traditional Chinese Medicine (Shanghai, China) due to being unconscious for half an hour. She had no previous history of symptoms such as headache, dizziness, blurred vision, and no strenuous exercise was performed before becoming unconsciousness. The patient was found to have a history of hypertension lasting 6 years, and her blood pressure at the time of admission to the hospital was 155/84 mmHg.

On physical examination, in addition to unclear consciousness, her Glasgow coma score was found to be 7 points, showing severe brain damage. The pupillary light reflex was normal, bilateral nasolabial sulci were symmetrical, and the tongue was in the middle while stretching. Physical examination of the heart, lung and abdomen was normal. She had lower muscle tension on both sides and positive pathological signs of both lower limbs.

Auxiliary examinations such as blood biochemistry and coagulation function showed Lp(a) 2100 mg/L (normal range 0–300); uric acid 201 µmol/L (normal range 210–430); low-density lipoprotein cholesterol 2.15 mmol/L (normal range 1.53–3.45); and d-dimer 1.26 µg/mL (normal range 0–0.2). The results of all routine diagnostic tests, such as blood, urine, stool and glycosylated haemoglobin, were normal.

Brain magnetic resonance imaging (MRI) suggested multiple acute cerebral infarctions in the vermis, cerebellar hemispheres and occipital lobes on both sides and multiple acute lacunar infarcts in the thalamus and left hippocampus on both sides. Brain magnetic resonance angiogram (MRA) showed irregular stenosis of the lumen of the right internal carotid artery, M1 and M2 segments of the right middle cerebral artery, and P2/P3 segments of the bilateral posterior cerebral artery. Intracranial computed tomography angiography (CTA) showed that the P2 branch of the bilateral posterior cerebellar artery and the left anterior inferior cerebellar artery diverged mostly in the form of soft plaque with varying degrees of luminal stenosis and right anterior inferior cerebellar artery thrombosis with embolism. The results of electrocardiography and colour ultrasound of the heart and abdomen were normal. Carotid artery ultrasound suggested right carotid artery plaque formation. The results of electrocardiography (ECG) and colour ultrasound of the heart and abdomen were found to be normal. Ultrasound of the carotid artery showed plaque formation in the right carotid artery.

The patient was diagnosed with ischaemic stroke due to basilar artery thrombosis. After admission, the patient was immediately treated with basilar artery stent thrombectomy, and the basilar artery responded well after the operation. Intracranial pressure was reduced by mannitol, platelet aggregation was prevented by aspirin and clopidogrel and the neurological symptoms were improved by edaravone. After 18 days of treatment, the patient showed improvement and was discharged.

Before discharge, her blood pressure was 140/70 mmHg, physical examination revealed that muscle strength of the 4 limbs was at level 5, and the Babinski and Chaddock signs on the left were positive. The National Institutes of Health Stroke Scale (NIHSS) score was 0 points. The patient could perform her daily routines and work during follow-up for 3 years. She had a clear consciousness, no aphasia, and no obvious abnormalities in cranial nerve examination. The muscle tension of the 4 limbs was normal, and the muscle strength was at level 5. The Lp(a) level of the patient continued to decrease to 875 mg/L through proper exercise and a reasonable diet.

**Fig. 1:**
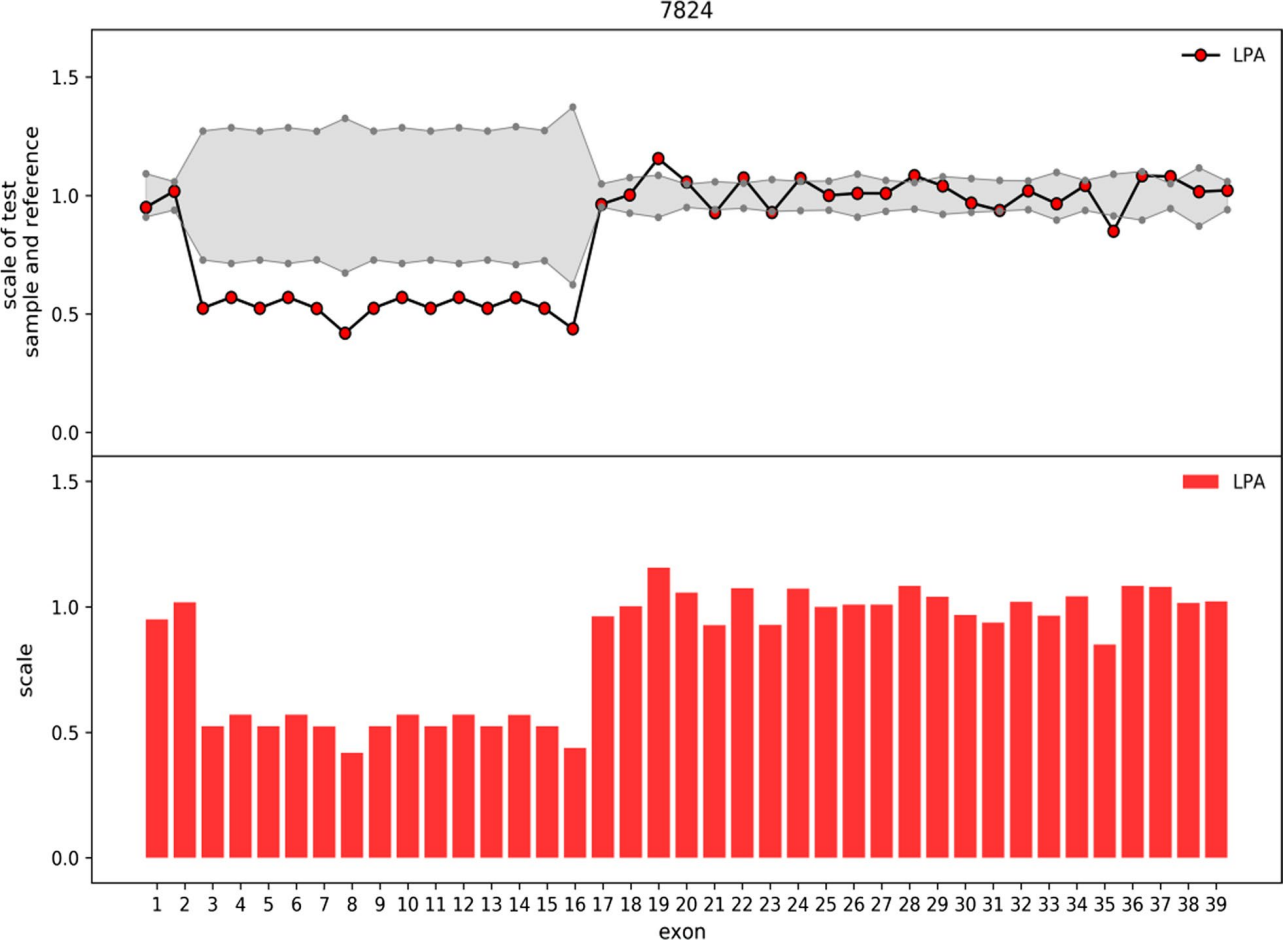
Identification of a heterozygous exonic deletion in the *LPA* gene of the patient. Genomic DNA extracted from external blood was subjected to NGS with a gene panel targeting 446 genes related to hereditary cardio-cerebrovascular diseases based on OMIM. Sequenced reads were mapped to the reference version of the human genome hg19 after the removal of the adapters and the low-grade reads. Large exon deletions and replications were screened by an internally developed overlay-based algorithm and eCNVscan. A heterozygous deletion of exons 3–16 covering KIV2 CNVs was identified

A total of 446 genes related to hereditary cardio-cerebrovascular diseases based on OMIM (Online Mendelian Inheritance in Man, http://omim.org) were included in the gene variation screening group of next-generation sequencing (NGS). In brief, genomic DNA extracted from external blood was segmented using Q800R Sonicator (Qsonica) to produce 300–500 bp insert fragments. Specially designed NimbleGen SeqCap probes (Roche NimbleGen, Madison, Wis) were used to hybridize in solution to enrich target sequences. A NextSeq500 sequencer (Illumina, San Diego, Calif) was used to index and sequence the enriched DNA samples with 100–150 cycles of single-end reads. After image analysis, initial data in FASTQ format was generated, and Illumina Pipeline was used for base calling. After the removal of the adapters and the low-grade reads, sequenced reads were mapped to the reference version of the human genome hg19 (release 2009-02, http://genome.ucsc.edu/). NextGENe software (SoftGenetics, State College, Pa) was used to call and review the observed nucleotide changes in the alignment sequences. An internally developed overlay-based algorithm and eCNVscan were used to screen for large exon deletions and replications. The standardized coverage depth of each exon in the experimental sample was in comparison with the average coverage depth of the same exon in the reference file. Population and literature databases containing 1000 Genomes, HGMD, gnomAD, ClinVar, dbSNP and OMIM, were used to annotate sequence variations [16]. Finally, targeted genetic analysis of the patient identified a novel heterozygous deletion of exons 3–16 covering KIV2 CNVs in the *LPA* gene (Fig. 1). This novel heterozygous deletion was further validated by qPCR using one set of primers detecting the second exon of KIV2 (KIV2 forward: 5′-CGACTATGCGAGTGTGGTGT-3′; KIV2 reverse: 5′-GGTGCAGGAGTGCTACCAT-3′). The 2-^ΔΔ^CT method with a normalized ratio to the reference gene (beta-actin) was used to conduct data analysis [17].

## Discussion and conclusions

In the current clinical case study, a Chinese female patient suffered from ischaemic stroke, and her plasma Lp(a) level was found to be significantly above the normal range. A novel compound heterozygous deletion covering KIV2 CNV in the *LPA* gene was found with genetic analysis through NGS and qPCR.

In a previous multiethnic study including South Asians, Chinese individuals, and European Caucasians, the genomic structure of the *LPA* locus was examined, and its contribution to Lp(a) concentrations was quantified. Both SNPs and KIV2 copy number are decisive factors of plasma Lp(a) concentrations, but relationships vary between ethnic groups. European Caucasians had the lowest copy number of KIV2, but it was most strongly correlated with plasma Lp(a) concentrations. SNPs may be involved in modifying the concentration of Lp(a) through the efficiency of transcription or expression [18].

Lu et al. performed a genome-wide association study to identify genetic variations associated with Lp(a)-cholesterol levels [19]. *LPA* sequencing was performed to further clarify the variation within *LPA*, and the two variants with the strongest correlation with Lp(a)-cholesterol were identified as rs3798220 and rs10455872. A significant correlation was found between the copy number of KIV2 and the level of Lp(a)-cholesterol. A lower copy number of KIV2 was associated with higher plasma Lp(a)-cholesterol levels. The number of KIV2 replicates was not only negatively correlated with the concentration of Lp(a) but also determined the size of the Lp(a) subtype. An increase in the number of KIV2 replicates led to an increase in the size of the Apo(a) subtype, which was associated with a low Lp(a) concentration [20]. In our study, a compound heterozygous deletion covering the KIV2 CNV in the *LPA* gene and high Lp(a) concentrations were related.

Lp(a) plays a vital role as a special lipoprotein carrier. Elevated Lp(a) levels may promote atherosclerosis through the inclusion of Lp(a)-derived cholesterol in the intima, recruitment of inflammatory cells, or binding to pro-inflammatory oxidizing phospholipids. The procoagulant and antifibrinolytic effects of apolipoprotein(a) on the one hand are manifested as inhibition of fibrinolysis and enhancement of coagulation stability, and on the other hand are manifested as enhancement of coagulation function through inhibition of tissue factor pathway inhibitors [21].

A cross-sectional study was performed in a Chinese Han population, and the Lp(a) concentrations were on average much lower than those in Caucasians. Elevated Lp(a) levels are a risk factor for coronary heart disease, and certain risk factors, such as primary hypertension, body mass index, total cholesterol, and creatinine, can alter Lp(a) levels [22]. It has also been recommended by one study to screen for a high level of Lp(a) in patients with a high or moderate risk of coronary heart diseases; less than 500 mg/L is an ideal level as a function of global cardiovascular risk [21]. Therefore, maintaining Lp(a) at a stable level is very important for the prevention of coronary heart disease.

A large-scale study revealed that elevated Lp(a) concentrations were related to increased acute myocardial infarction risk in a Chinese population with normal LDL cholesterol levels [23]. A high level of Lp(a) may promote the formation of atherosclerosis, which leads to an increased risk of cerebral thrombosis. In a population-based cohort study, Lp(a) concentrations were higher in black participants than in white participants and higher in women than in men. Elevated Lp(a) was associated with an increased risk of ischaemic stroke. Although the racial difference was not statistically significant, this trend was more pronounced among black participants than white participants. Lp(a) values were significantly lower in men than in women, but the association between Lp(a) and ischaemic stroke did not differ between men and women. The use of statins did not change the relationship between Lp(a) and stroke [24]. The concentration of Lp(a) varies among ethnic groups, but its association with cardiovascular and cerebrovascular diseases is not different. In this report, the Lp(a) level of the patient was highly increased (2100 mg/L), which suggests that elevated Lp(a) was related to the occurrence of ischaemic stroke.

In conclusion, our study is generally consistent with previous research. The current study may suggest that the genetic variants in *LPA* of the patient were likely to be correlated with an increased level of Lp(a) and the occurrence of ischaemic stroke. Our case has some implications for the prevention and treatment of ischaemic stroke. However, a detailed study is required to identify the specific mechanism of *LPA* mutations in ischaemic stroke.

## Data Availability

The datasets generated and analysed during the current study are available in the NCBI Sequence Read Archive database (accession number: SRR14381436, https://www.ncbi.nlm.nih.gov/sra/?term=SRR14381436). The web links of the relevant datasets were as follows: hg19 (http://genome.ucsc.edu/), OMIM (http://omim.org), 1000 Genomes (https://www.internationalgenome.org/), dbSNP (http://www.bioinfo.org.cn/relative/dbSNP%20Home%20Page.htm), gnomAD (https://gnomad.broadinstitute.org/about), ClinVar (https://www.ncbi.nlm.nih.gov/clinvar/), HGMD (http://www.hgmd.cf.ac.uk/ac/index.php).

## Abbreviations

Lp(a): Lipoprotein(a)
LDL: Low-density lipoprotein
apo(a): Apolipoprotein(a)
PLG: Plasminogen
CNVs: Copy number variations
CVD: Cardiovascular disease
CAHD: Coronary atherosclerotic heart disease
MRI: Magnetic resonance imaging
MRA: Magnetic resonance angiogram
CTA: Computed tomography angiography
ECG: Electrocardiograph
NGS: Next-generation sequencing
SNPs: Single nucleotide polymorphisms

## References

[1] Schmidt K, Noureen A, Kronenberg F, Utermann G (2016). Structure, function, and genetics of lipoprotein (a). J Lipid Res.

[2] Frank SL, Klisak I, Sparkes RS, Mohandas T, Tomlinson JE, McLean JW, Lawn RM, Lusis AJ (1988). The apolipoprotein(a) gene resides on human chromosome 6q26-27, in close proximity to the homologous gene for plasminogen. Hum Genet.

[3] Haibach C, Kraft HG, Köchl S, Abe A, Utermann G (1998). The number of kringle IV repeats 3–10 is invariable in the human apo(a) gene. Gene.

[4] McLean JW, Tomlinson JE, Kuang WJ, Eaton DL, Chen EY, Fless GM, Scanu AM, Lawn RM (1987). cDNA sequence of human apolipoprotein(a) is homologous to plasminogen. Nature.

[5] Utermann G (1989). The mysteries of lipoprotein(a). Science.

[6] Sandholzer C, Hallman DM, Saha N, Sigurdsson G, Lackner C, Császár A, Boerwinkle E, Utermann G (1991). Effects of the apolipoprotein(a) size polymorphism on the lipoprotein(a) concentration in 7 ethnic groups. Hum Genet.

[7] Gaw A, Boerwinkle E, Cohen JC, Hobbs HH (1994). Comparative analysis of the apo(a) gene, apo(a) glycoprotein, and plasma concentrations of Lp(a) in three ethnic groups. Evidence for no common "null" allele at the apo(a) locus. J Clin Invest.

[8] Kraft HG, Lingenhel A, Pang RW, Delport R, Trommsdorff M, Vermaak H, Janus ED, Utermann G (1996). Frequency distributions of apolipoprotein(a) kringle IV repeat alleles and their effects on lipoprotein(a) levels in Caucasian, Asian, and African populations: the distribution of null alleles is non-random. Eur J Hum Genet.

[9] Virani SS, Brautbar A, Davis BC, Nambi V, Hoogeveen RC, Sharrett AR, Coresh J, Mosley TH, Morrisett JD, Catellier DJ (2012). Associations between lipoprotein(a) levels and cardiovascular outcomes in black and white subjects: the Atherosclerosis Risk in Communities (ARIC) Study. Circulation.

[10] Kraft HG, Köchl S, Menzel HJ, Sandholzer C, Utermann G (1992). The apolipoprotein (a) gene: a transcribed hypervariable locus controlling plasma lipoprotein (a) concentration. Hum Genet.

[11] Clarke R, Peden JF, Hopewell JC, Kyriakou T, Goel A, Heath SC, Parish S, Barlera S, Franzosi MG, Rust S (2009). Genetic variants associated with Lp(a) lipoprotein level and coronary disease. N Engl J Med.

[12] Danesh J, Collins R, Peto R (2000). Lipoprotein(a) and coronary heart disease. Meta-analysis of prospective studies. Circulation.

[13] Erqou S, Kaptoge S, Perry PL, Di Angelantonio E, Thompson A, White IR, Marcovina SM, Collins R, Thompson SG, C Emerging Risk Factors (2009). Lipoprotein(a) concentration and the risk of coronary heart disease, stroke, and nonvascular mortality. JAMA.

[14] Sun L, Zong M, Chen C, Xie L, Wu F, Yu M, Fan L (2018). Low LPA gene kringle IV-2 repeat copy number association with elevated lipoprotein (a) concentration as an independent risk factor of coronary atherosclerotic heart disease in the Chinese Han population. Lipids Health Dis.

[15] Nave AH, Lange KS, Leonards CO, Siegerink B, Doehner W, Landmesser U, Steinhagen-Thiessen E, Endres M, Ebinger M (2015). Lipoprotein (a) as a risk factor for ischemic stroke: a meta-analysis. Atherosclerosis.

[16] Ge T, Zhang X, Xiao Y, Wang Y, Zhang T (2019). Novel compound heterozygote mutations of TJP2 in a Chinese child with progressive cholestatic liver disease. BMC Med Genet.

[17] Kamstrup PR, Tybjaerg-Hansen A, Steffensen R, Nordestgaard BG (2009). Genetically elevated lipoprotein(a) and increased risk of myocardial infarction. JAMA.

[18] Lanktree MB, Anand SS, Yusuf S, Hegele RA, Investigators S (2010). Comprehensive analysis of genomic variation in the LPA locus and its relationship to plasma lipoprotein(a) in South Asians, Chinese, and European Caucasians. Circ Cardiovasc Genet.

[19] Lu W, Cheng YC, Chen K, Wang H, Gerhard GS, Still CD, Chu X, Yang R, Parihar A, O'Connell JR (2015). Evidence for several independent genetic variants affecting lipoprotein (a) cholesterol levels. Hum Mol Genet.

[20] Mu-Han-Ha-Li DL, Zhai TY, Ling Y, Gao X (2018). LPA kringle IV type 2 is associated with type 2 diabetes in a Chinese population with very high cardiovascular risk. J Lipid Res.

[21] Nordestgaard BG, Chapman MJ, Ray K, Borén J, Andreotti F, Watts GF, Ginsberg H, Amarenco P, Catapano A, Descamps OS (2010). Lipoprotein(a) as a cardiovascular risk factor: current status. Eur Heart J.

[22] Cai DP, He YM, Yang XJ, Zhao X, Xu HF (2015). Lipoprotein (a) is a risk factor for coronary artery disease in Chinese Han ethnic population modified by some traditional risk factors: a cross-sectional study of 3462 cases and 6125 controls. Clin Chim Acta.

[23] Cai G, Huang Z, Zhang B, Yu L, Li L (2019). Elevated lipoprotein (a) levels are associated with the acute myocardial infarction in patients with normal low-density lipoprotein cholesterol levels. Biosci Rep..

[24] Arora P, Kalra R, Callas PW, Alexander KS, Zakai NA, Wadley V, Arora G, Kissela BM, Judd SE, Cushman M (2019). Lipoprotein(a) and risk of ischemic stroke in the REGARDS study. Arterioscler Thromb Vasc Biol.

